# Anaphylaxis in the emergency room in the city of São Paulo: do we know how to manage?

**DOI:** 10.1186/1939-4551-8-S1-A129

**Published:** 2015-04-08

**Authors:** Cynthia Lima

**Affiliations:** 1 Universidade Anhembi Morumbi, Brazil

## Background

Evaluate the knowledge of physicians who work in emergency services in the City of São Paulo on terms, management and conditions related to anaphylaxis.

## Methods

Descriptive study. After signing the term of free and informed consent, physicians who work in emergency services in the city of São Paulo were asked to answer a questionnaire.

## Results

Out of 177 physicians invited to take part of the survey, only 77 accepted. Out of which, 48,1% have already treated an anaphylactic reaction. Eventhough 49% deny ever having attended a case, they claim having treated of alergy set on more than one organic system.

As a non-pharmacologic measure, the most mentioned was oxygen. As a pharmacologic measure, adrenalin was the choice of 69% of physicians, also mentioning the intramuscular pathway only by 51,85%. Corticosteroids were chosen as a consecution for the initial therapy by 19,6%. Glucagon was elected by four physicians as an alternative therapy for the management of a refractory anaphylactic reaction in patients in cronic use of beta blockers. Out of the 77 doctors, 16 (20,77%) already heard of biphasic anaphylactic reactions, while 79,23% had no acknowledgement.

## Conclusions

49% of the interviewee proved not knowing the actual criteria for diagnosing anaphylaxis. As the late diagnosis diminishes the survival rate of these patients, it is important to elevate the knowledge on this subject for doctors working on emergency services.

